# Panel Fullness, Burnout, and Intention to Leave Among Veterans Health Administration Primary Care Practitioners

**DOI:** 10.1001/jamanetworkopen.2026.33671

**Published:** 2026-09-15

**Authors:** David C. Mohr, Robert C. Oh, Michael E. Charness

**Affiliations:** 1 National Center for Organization Development, Veterans Health Administration, Mason, Ohio; 2 Department of Health Law, Policy & Management, Boston University School of Public Health, Boston, Massachusetts; 3 Palo Alto Health Care System, Department of Veterans Affairs, Palo Alto, California; 4 Department of Medicine and Division of Primary Care and Population Health, Stanford University School of Medicine, Stanford, California; 5 VA Boston Healthcare System, West Roxbury, Massachusetts; 6 Department of Neurology, Harvard Medical School, Boston, Massachusetts; 7 Department of Neurology, Boston University School of Medicine, Boston, Massachusetts; 8 VA Boston Healthcare System, Boston, Massachusetts

## Abstract

**Importance:**

Primary care practitioners (PCPs) experience high levels of burnout despite mitigation efforts. The foundational nature of primary care in the US health care system obligates a better understanding of the root causes of burnout among PCPs.

**Objective:**

To determine whether an association exists between patient panel fullness and PCP burnout and intention to leave.

**Design, Setting, and Participants:**

In this cross-sectional study, annual Veterans Health Administration data from 2022 to 2024 were reviewed for primary care panel fullness, support staffing, and clinic characteristics. Participants included all PCP (physicians, nurse practitioners, and physician assistants) respondents to the Veterans Administration (VA) All Employee Survey between 2022 and 2024.

**Main Outcomes and Measures:**

The primary outcomes were PCP burnout and intention to leave the VA derived from responses to the annual All Employee Survey. Data were aggregated across 139 facilities and analyzed using cross-sectional and year-to-year change regression models.

**Results:**

The study had 15 856 survey respondents across 139 medical centers over 3 years, with response rates of 69.9% in 2022, 74.3% in 2023, and 74.1% in 2024. Of them, 16.4% were younger than 40 years, 26.5% were 40 to 49 years of age, 32.6% were 50 to 59 years, and 20.8% were 60 years or older; 54.2% self-reported being female. Across that time, the mean (SD) burnout rate for PCPs was 55.2% (12.6%), and it was 31.9% (13.2%) for intention to leave the VA. Panel fullness increased from 86.3% in 2022 to 92.8% in 2024. In cross-sectional models, panel fullness was associated with higher rates of burnout (odds ratio [OR], 1.07 [95% CI, 1.00-1.13]) and intention to leave (OR, 1.09 [95% CI, 1.01-1.18]). Year-to-year increases in panel fullness were associated with higher intention to leave (OR, 1.13 [95% CI, 1.01-1.25]), as was prior year panel fullness (OR, 1.19 [95% CI, 1.07-1.33]). Support staff per PCP ratio was associated with higher burnout (OR, 1.32 [95% CI, 1.10-1.58]).

**Conclusions and Relevance:**

In this cross-sectional study of PCPs at the VA, increased patient panel fullness was associated with increased PCP burnout and intention to leave. These findings suggest that organizations seeking to increase primary care capacity should weigh the potential downsides of increasing patient panel fullness or capacity.

## Key Points

**Question:**

Is high patient panel fullness associated with burnout and intention to leave among primary care practitioners?

**Findings:**

In a cross-sectional study of 15 856 survey respondents across 139 facilities, 55% of Veterans Administration primary care practitioners reported burnout and 32% reported their intention to leave. High patient panel fullness was associated with both higher burnout and intention to leave.

**Meaning:**

These findings suggest that high patient panel fullness may worsen primary care burnout and increase intention to leave.

## Teaser

This cross-sectional study investigates whether an association exists between patient panel fullness and Veterans Administration primary care practitioner burnout and intention to leave.

## Introduction

Burnout of health care professionals surged during the COVID-19 pandemic and then decreased to a high plateau.^1^ While mitigation efforts have helped reverse overall national physician burnout trends, burnout remains high for primary care physicians.^1,2,3^ Primary care physician burnout rates in the Veterans Health Administration (VHA) rose from 46% in 2018 to 58% in 2022, a rate much higher than for other specialties.^2^ Burnout among Veterans Administration (VA) and non-VA primary care physicians is associated with intention to leave.^4,5^ The foundational nature of primary care in the US health care system and a growing national shortage of primary care physicians highlight the importance of understanding and addressing the root causes of their burnout.

The root causes of burnout in health care have been analyzed in a framework with 2 principal components: job demands, including workload, time pressure, emotional strain, and task complexity; and job resources, including autonomy, support, recognition, and skill development.^6^ A critical element of job demands is the number of patients in a panel, and a critical element of job resources is primary care practitioner (PCP) support staff. In the VHA, the primary care Patient Aligned Care Team (PACT) comprises a PCP (physician, advanced practice nurse, or physician assistant), a registered nurse care coordinator, a health technician or licensed practical nurse, and a medical support assistant for scheduling. The PACT panel size is determined by a variety of factors, including PCP profession (physician vs advanced practice practitioner), patient complexity, examination room availability, and support staffing.^7^

A cross-sectional survey of selected VA primary care practices prior to the COVID-19 pandemic identified burnout in 41% of PACTs.^8^ Higher rates of burnout were associated with poor staffing, team turnover, and overempanelment.^8^ Support staffing may be a mitigating factor for burnout by offloading clinical and administrative tasks from PCPs to nursing and administrative staff.^9^ Likewise, changes in job demands, such as changing scope and increasing volume of work, may impact employee burnout and intention to leave.^10,11,12^

In 2022, the Promise to Address Comprehensive Toxics Act was signed into law and was one of the largest expansions of health care eligibility for the VHA.^13^ In anticipation of a large influx of new VA enrollees, the VHA implemented the Access Sprint initiative in fall and winter of 2023 to increase primary care access, in part by increasing panel fullness to 95% to 105% of calculated panel size. This change afforded an opportunity to evaluate the association of increases in panel fullness on burnout and intention to leave.

In the current study, we examined data from national VA surveys to characterize burnout, intention to leave, job satisfaction, and perception of reasonable workload between 2022 and 2024. We tested the hypotheses that high panel fullness is associated with high burnout, increased intention to leave, reduced job satisfaction, and decreased perception that the workload is reasonable and evaluated the association of factors of risk and resilience with these outcome measures.

## Methods

### Study Design

This cross-sectional study involved a repeated-measures observational design using secondary data from the VA. The objective was to analyze the influence and change in patient panel fullness and support staff on clinician workplace experiences. Data were obtained from administrative databases along with employee responses from an annual organization-wide survey. The scope of work for the study was reviewed by the VA Palo Alto institutional review board and Stanford University as human participants research and was determined to meet the exemption criteria for nonresearch activities and received a waiver for ethical review. The board also granted a waiver of informed consent because the study did not meet the criteria of human participants research. The Strengthening the Reporting of Observational Studies in Epidemiology (STROBE) guideline for cross-sectional studies was followed.

### Sample and Instrument

The sample consisted of VA employees who responded to the All Employee Survey (AES) conducted in the spring of 2022, 2023, and 2024. The AES was administered to all employees across the Department of Veterans Affairs on an annual basis to understand employee perceptions of the workplace and to achieve organizational improvement.^14^ It is administered by an independent vendor and allows for multiple methods of completion, with more than 99% of respondents providing responses online. To identify PCPs, we selected respondents who self-reported their occupation as a physician, nurse practitioner, or physician assistant and indicated that they were currently a member of a PACT.

### Measures

Our outcomes of interest were obtained from responses to the AES. Burnout and intention to leave were our primary outcomes, and workload and job satisfaction were secondary outcomes. *Burnout* was defined based on 2 items from the Maslach Burnout Inventory.^15^ The items asked about emotional exhaustion (“I feel burned out from my work”) and depersonalization (“I worry that this job is hardening me emotionally”). Respondents indicated how often they felt that way using a 7-point scale (0 for never to 6 for every day). Items were examined for reliability and validity in context of the full instrument.^16,17^ PCPs who indicated that they felt that way once or more a week to either item were coded as experiencing burnout, as this is a commonly used threshold in prior research. *Turnover intention* was coded based on responses of either “no” or “yes” along with the reasons that they were considering leaving their job within the next year. We also modeled *reasonable workload* with a single item: “My workload is reasonable,” which was dichotomized to represent responses of strongly agree and agree compared with disagreement or neutral responses. This approach also served as a test of whether an objective workload measure would relate to subjective ratings. We also modeled *job satisfaction* based on a dichotomously coded item of “Considering everything, how satisfied are you with your job?” Responses of satisfied or very satisfied were compared with responses of very dissatisfied, dissatisfied, and neutral.

Metrics to represent facility-level panels were obtained from the VA’s Patient Aligned Care Team Compass. *Panel fullness* was defined as the total number of active patient assignments to a team divided by the number of patients that can be assigned. *Support staff per PCP ratio* was defined as the total support staff full-time employees divided by the total number of PCP full-time employees. Support staff included registered nurse care managers, clinical associates (including licensed practical nurses, health technicians, or medical assistants), or administrative associates working within the primary care clinic. Additional site-level characteristics were included in the model to adjust for factors that can potentially influence burnout and intention to leave. We included annual measures of the mean age of patients and the panel revisit rate, defined as the mean number of primary care encounters per active patients on the panel. We modeled the traditional encounters ratio as the number of in-person visits compared with the total number of visits, which included in-person visits and telephone, video, group appointments, and secure messaging encounters. Higher values reflected greater face-to-face care. We accounted for clinic size using the number of PCP patient visits in units of 10 000 patients. We included medical center variables that represented rurality, coded as either urban or rural; geographic region, defined based on the 4 US Census Regions; and whether the medical center was a historic member of the Council of Teaching Hospitals (COTH) (scored 0 or 1), indicating a higher level of affiliation and teaching activity. We also accounted for temporal patterns by including the study year.

### Statistical Analysis

We first examined data for missing and out of range values and confirmed completeness of cases when merging multiple datasets. Multicollinearity was examined among variables. We examined burnout and intention to leave for PCPs. For panel fullness and the outcome variables, we examined whether there were significant differences per year among responding PCPs at medical centers using 1-way analysis of variance. We report 2 types of model results using facility-level data. First, we conducted cross-sectional models to examine whether panel fullness in the same year was associated with clinician outcomes. We then examined whether year-to-year changes in panel fullness were associated with clinician outcomes. In these models, we controlled for prior-year fullness (time 2 minus time 1). The key independent variable in this model was the change in panel fullness from the prior year, scaled so that 1 unit represented a 10–percentage point increase.

We modeled outcome, such as burnout, as a clustered binary outcome using generalized linear mixed-effects models. Because the analysis used medical center–level data, we specified outcomes as number of PCPs reporting a positive value for the outcome (ie, burnout) per total number of PCP respondents at the medical center using a binomial distribution. Medical center was included as a random-effects model. Using generalized linear mixed-effects models, we also examined the estimate of prior-year panel fullness on clinician burnout using a similar set of measures. We created a lagged variable for panel fullness per 10% increase in prior-year fullness. This approach isolated within-facility change and reduced bias that may occur when some facilities have little or no change. We used a random intercept for medical center. We estimated models using maximum likelihood with Laplace approximation and robust standard errors for fixed effects. We examined model diagnostics to assess model fit. Overdispersion was examined as χ^2^-to-*df* ratios, and all values were less than 1. Random intercept variance for the medical center was nonzero, and all models converged normally without boundary estimates. Missing data were rare (3 out of 418 facility-year observations). The analysis used available case-methods. Year (referent of 2022) was included as a fixed effect in cross-sectional models. For change score models, we used 2024 minus 2023 compared with 2023 minus 2022. We report odds ratios (ORs) with 95% CIs. In sensitivity analyses, we classified panel fullness into categories (fullness <85%, 85% to <95%, 95% to <105%, and ≥105%). We examined categorical analysis of facilities that had higher than 5% increases, 5% decreases, or were within 5% change in panel fullness and compared any increase in panel fullness with any decrease or no change. We examined whether video, telephone, or secure message visit rate influenced outcomes. We examined a conservative coding of burnout as occurring a few times a week or every day and as a continuous measure.

SAS, version 9.4 (SAS Institute Inc) was used for analysis. A 2-sided *P* < .05 was considered statistically significant.

## Results

The survey had 15 856 PCP respondents, with 4923 (2022), 5404 (2023), and 5529 (2024) respondents for each year across 139 medical centers and response rates of 69.9%, 74.3%, and 74.1%, respectively. Of them, 16.4% were younger than 40 years, 26.5% were 40 to 49 years of age, 32.6% were 50 to 59 years, and 20.8% were 60 years or older; 54.2% were female, 31.9% were male, and 13.9% missing or not reported. After assessing item properties and merging datasets, we examined descriptive statistics means over the study period (Table 1) as well as annually and by change by year for key measures (eTable in Supplement 1).

**Table 1.** Descriptive Statistics for Mean Annual Values of Study Variables at the Medical Center Level (N = 139) for PCPs, 2022 Through 2024

Measure	Value
Burnout, mean (SD)^a^	0.55 (0.12)
Intention to leave, mean (SD)^a^	0.32 (0.13)
Reasonable workload, mean (SD)^a^	0.63 (0.14)
Job satisfaction, mean (SD)^a^	0.62 (0.14)
Panel fullness, mean (SD)^a^	0.89 (0.09)
Patient age, mean (SD), y	62.84 (2.83)
Panel revisit rate^b^	2.14 (4.7)
Traditional encounter rate^c^	0.55 (0.14)
Support staff per PCP, mean (SD)	2.66 (.39)
PCP visits per 10 000 patients, mean (SD)	5.28 (3.30)
Rural facility, No. (%)	17 (12.2)
COTH Member No. (%)^d^	72 (51.8)
Geographic region No. (%)	
Northeast	26 (18.7)
Midwest	34 (24.5)
South	50 (36.0)
West	29 (20.9)

Abbreviations: COTH, Council of Teaching Hospital; PCP, primary care practitioner.

^a^ Based on the mean value of the responses from PCPs at medical centers to the item; each measure scored as 0 for absent or 1 for present.

^b^ Panel revisit rate reflects mean number of patient visits per year.

^c^ Traditional encounters represent rate of in-person visits per total.

^d^ COTH membership was used as a proxy for more intense teaching activity.

Panel fullness increased from 86.3% to 86.7% to 92.8% across 3 consecutive years (*P* < .001). The support staff per PCP ratio increased incrementally over this time, from 2.58 to 2.62 to 2.77 (*P* < .001). Among 139 medical centers for all 3 years, we found that the mean (SD) burnout was 55.2% (12.6%), intention to leave was 31.9% (13.2%), job satisfaction was 62.1% (14.0%), and workload satisfaction was 43.5% (14.1%). More than half of the medical centers (72 [51.8%]) were members of COTH, and 17 (12.2%) were in rural areas.

Annual values for study outcomes were plotted against patient panel fullness to show patterns (Figure). In cross-sectional models, higher panel fullness was associated with higher burnout (OR, 1.07 [95% CI, 1.00-1.13]; *P* = .04) and intention to leave (OR, 1.09 [95% CI, 1.01-1.18]; *P* = .03) rates and lower ratings on reasonable workload (OR, 0.90 [95% CI, 0.84-0.97]; *P* < .001) (Table 2). More support staff per PCP was associated with higher burnout (OR, 1.19 [95% CI, 1.03-1.38]), with the year-to-year change in support staff per PCP ratio also associated with higher burnout (OR, 1.32 [95% CI, 1.10-1.58]). Among the covariates, COTH membership was associated with favorable PCP-reported outcomes, including less burnout (OR, 0.87 [95% CI, 0.77-0.99]), favorable perceptions of a reasonable workload (OR, 1.30 [95% CI, 1.13-1.50]), and higher job satisfaction (OR, 1.31 [95% CI, 1.11-1.54]). Higher panel revisit rate was also associated with lower intention to leave (OR, 0.73 [95% CI, 0.61-0.86]) and higher job satisfaction (OR, 1.30 [95% CI, 1.09-1.56]). A greater proportion of traditional in-person encounters was associated with lower burnout (OR, 0.48 [95% CI, 0.30-0.79]) and with higher perceptions that the workload was reasonable (OR, 2.04 [95% CI, 1.21-3.43]).

**Figure.**
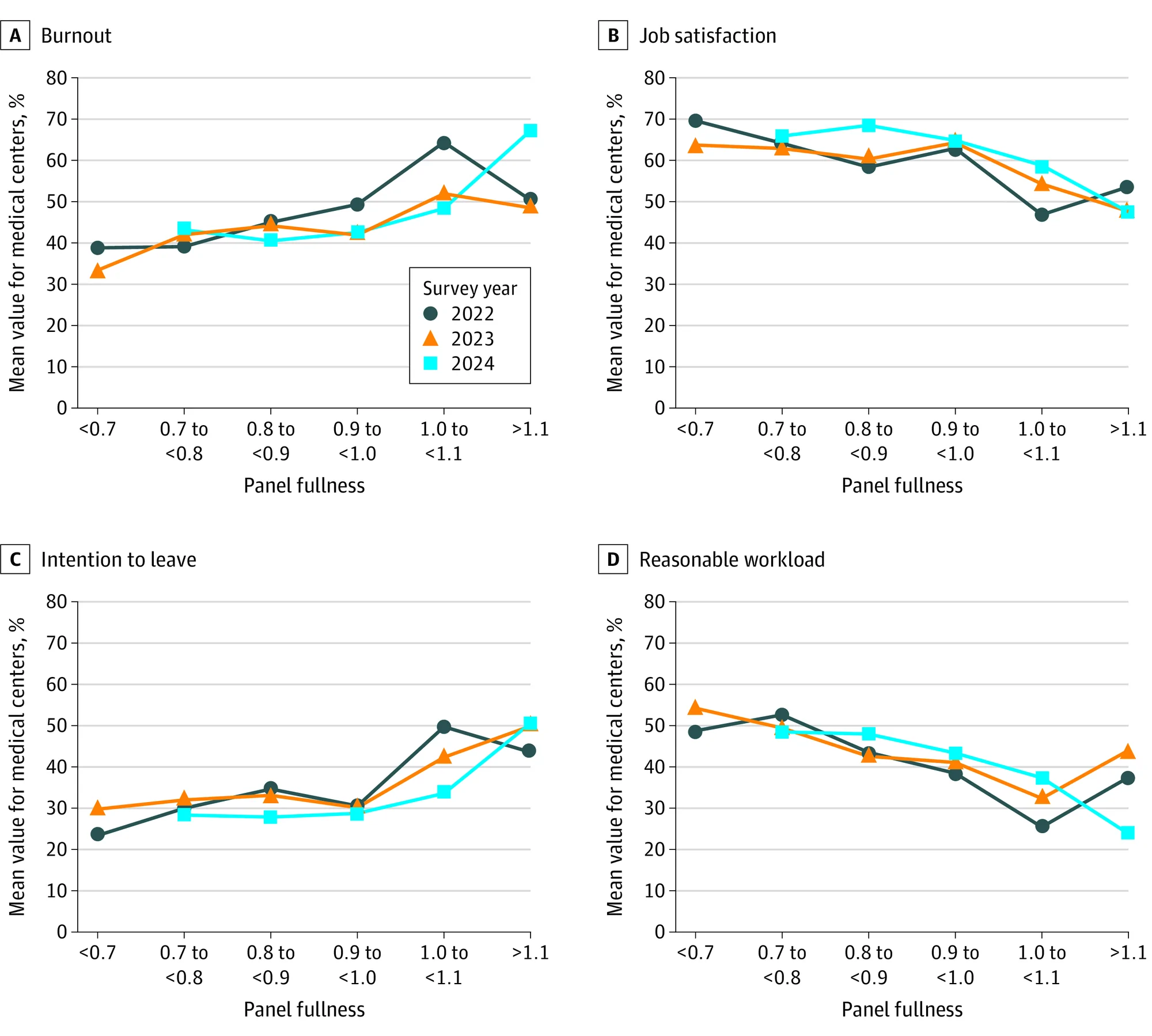
Line Graphs Illustrating the Association Between Panel Fullness and Employee Outcomes Across 3 Years Higher values indicate greater levels of the respective outcome.

**Table 2.** Cross-Sectional Models for Panel Fullness and PCP Job Experiences (139 Facilities Repeated 3 Years)^a^

Variable	Burnout		Intention to leave		Reasonable workload		Job satisfaction	
	OR (95% CI)	*P* value	OR (95% CI)	*P* value	OR (95% CI)	*P* value	OR (95% CI)	*P* value
Patient panel fullness per 10%	1.07 (1.00-1.13)	.04	1.09 (1.01-1.18)	.03	0.90 (0.84-0.97)	<.001	0.94 (0.88-1.00)	.06
Support staff per PCP	1.19 (1.03-1.38)	.02	1.01 (0.86-1.20)	.89	.088 (0.74-1.03)	.11	.94 (0.81-1.10)	.46
Mean patient age	1.00 (0.98-1.02)	<.001	1.00 (0.97-1.03)	.86	1.00 (0.98-1.03)	.74	1.01 (0.98-1.05)	.37
Panel revisit rate^b^	0.88 (0.75-1.03)	.10	0.73 (0.61-.86)	<.001	1.15 (0.93-1.42)	.19	1.30 (1.09-1.56)	.61
Traditional encounters^c^	0.48 (0.30-0.79)	.003	0.77 (0.41-1.42)	.40	2.04 (1.21-3.43)	.008	1.44 (0.78-2.66)	.24
PCP visits per unique patients	1.00 (0.99-1.02)	.60	0.98 (0.96-1.00)	.10	0.99 (.97-1.01)	.21	1.00 (0.98-1.01)	.61
COTH member^d^	0.87 (0.77-0.99)	.04	0.86 (0.73-1.02)	.09	1.30 (1.13-1.50)	<.001	1.31 (1.11-1.54)	.001
Rural	0.93 (0.74-1.17)	.54	1.15 (0.89-1.49)	.28	0.95 (.75-1.19)	.62	0.88 (.67-1.14)	.32
Year								
2023 vs 2022	0.84 (0.75-0.94)	.002	.98 (0.85-1.12)	.72	1.15 (1.03-1.29)	.01	1.13 (1.00-1.28)	.05
2024 vs 2022	0.74 (0.64-0.86)	<.001	0.81 (0.69-0.95)	.01	1.36 (1.17-1.58)	<.001	1.37 (1.17-1.59)	<.001

Abbreviations: COTH, Council of Teaching Hospital; OR, odds ratio; PCP, primary care practitioner.

^a^ Geographic region included but not represented in output.

^b^ Panel revisit rate reflects mean number of patient visits per year; higher number of visits relates to lower likelihood intention to leave in this table.

^c^ Traditional encounters represent rate of in-person visits per total visits; higher nontraditional values relate to higher likelihood of burnout in this table.

^d^ COTH membership was used as a proxy for more intense teaching activity.

Year-to-year increases in patient panel fullness were associated with higher intention to leave (OR, 1.13 [95% CI, 1.01-1.25]). Prior year panel fullness was associated with lower perception that the workload was reasonable (OR, 0.86 [95% CI, 0.79-0.93]) and greater intention to leave (OR, 1.19 [95% CI, 1.07-1.33]) (Table 3). A comparison of 2024 to 2023 revealed decreases in burnout (OR, 0.89 [95% CI, 0.80-0.89]) and intention to leave (OR, 0.83 [95% CI, 0.74-0.93]) and increases in job satisfaction (OR, 1.21 [95% CI, 1.08-1.36]) and perception that the workload was reasonable (OR, 1.14 [95% CI, 1.01-1.29]).

**Table 3.** Year-to-Year Changes in Panel Fullness and PCP Job Experiences (139 Facilities Across 2 Years)^a^

Variable	Prior year change							
	Burnout		Intention to leave		Reasonable workload		Job satisfaction	
	OR (95% CI)	*P* value	OR (95% CI)	*P* value	OR (95% CI)	*P* value	OR (95% CI)	*P* value
Patient panel fullness: change	1.02 (0.93-1.11)	.20	1.13 (1.01-1.25)	.04	0.95 (0.85-1.07)	.40	0.93 (0.83-1.04)	.20
Patient panel fullness: prior year	1.08 (0.99-1.17)	.06	1.19 (1.07-1.33)	.002	0.86 (0.79-0.93)	<.001	0.90 (0.81-1.00)	.06
Support staff per PCP	1.32 (1.10-1.58)	.47	0.98 (0.78-1.23)	.84	0.86 (0.70-1.06)	.16	0.92 (0.73-1.16)	.47
Mean patient age	1.00 (0.98-1.02)	.30	1.00 (0.96-1.04)	.98	1.01 (0.98-1.03)	.64	1.02 (0.98-1.06)	.30
Panel revisit rate^b^	0.86 (0.72-1.03)	.07	0.77 (0.63-0.95)	.01	1.23 (0.97-1.58)	.09	1.25 (0.98-1.59)	.07
Traditional encounters^c^	0.43 (0.24-0.75)	.39	0.81 (0.42-1.57)	.54	2.11 (1.16-3.84)	.01	1.38 (0.66-2.90)	.39
PCP visits per unique patients	1.00 (0.99-1.02)	.99	0.98 (0.96-1.00)	.04	1.00 (0.98-1.02)	.64	1.00 (0.97-1.03)	.99
COTH member^d^	0.88 (0.76-1.03)	.03	0.94 (0.77-1.15)	.56	1.26 (1.08-1.48)	.005	1.23 (1.02-1.49)	.03
Rural	0.95 (0.71-1.27)	.18	1.22 (.90-1.66)	.20	0.95 (0.72-1.25)	.68	0.82 (0.61-1.10)	.18
Change 2024 to 2023 vs 2023 to 2022	0.89 (0.80-0.99)	.001	0.83 (0.74-0.93)	.001	1.14 (1.01-1.29)	.04	1.21 (1.08-1.36)	.001

Abbreviations: COTH, Council of Teaching Hospital; OR, odds ratio; PCP, primary care practitioner.

^a^ Model examines the change from 2023 to 2024 and 2022 to 2023. Geographic region included but not represented in output.

^b^ Panel revisit rate reflects mean number of patient visits per year, which indicates lower odds of intention to leave in this table.

^c^ Traditional encounters represent rate of in-person visits per total visits; higher nontraditional encounters indicate higher odds of burnout in this table.

^d^ COTH membership was used as a proxy for more intense teaching activity.

In sensitivity analyses using a categorical model, when panel fullness was 105% or greater, relative to panel fullness of 85% to less than 95%, the perception that the workload was reasonable was lower (OR, 0.77 [95% CI, 0.61-0.96]). Most analysis of variance comparisons were not statistically significant when looking at whether panel fullness increased, decreased, or remained stable and whether there was any increase at all. Although the direction of change aligned with the primary findings, the secondary outcome of reasonable workload showed a statistically significant difference across categories. Further, patient panel fullness per 10% increment was still associated with burnout when the more conservative dichotomous criteria (burnout experienced a few times per week or every day) were applied (OR, 1.10 [95% CI, 1.03-1.16]) and when analyzing burnout as a continuous measure (*b* = 0.08, SE = 0.04, *P* = .04).

## Discussion

An important finding of this cross-sectional study investigating whether patient panel fullness is associated with PCP burnout and turnover intention was that VA PCPs experienced high rates of burnout. The burnout rate for VA PCPs on PACTs was 55.6% in 2024. A previous study reported a burnout rate of 57.6% among VA primary care physicians in 2022.^2^ National data from the American Medical Association demonstrated a decline in burnout rates over the same period, from 53.0% to 43.2% for all physicians and from 51.4% to 42.0% for internal medicine physicians.^18^ This reduction in burnout since 2022 likely reflects emergence from the COVID-19 pandemic and the implementation of measures to reduce job demands and increase job resources, including reduced administrative burdens, increased operational efficiency, and improved physician resilience.^3^ The relative resistance of burnout to these measures in VA PCPs is concerning and merits closer evaluation since burnout in the present study and prior publications was associated with intention to leave.^4^ Intention to leave was higher in VA PCPs in our study (31.9%) than for VA PCPs surveyed from 2017 to 2021 (6.3%-8.4%)^19^ or for physicians from an urban academic medical center surveyed in 2022 (13.5%).^9^ Within the VHA, higher burnout was also associated with increased turnover,^4,19^ and the cost of turnover to an organization was estimated at $0.5 to $1.0 million per physician.^20^

Cross-sectional analysis minimizes the confounding of events that change the trajectory of burnout over time, such as emergence from the COVID-19 pandemic and the implementation of measures to mitigate burnout. In our cross-sectional analysis, higher patient panel fullness was associated with increased burnout, greater intention to leave, and decreased perception that the workload was reasonable. The association of panel fullness with the perception of reasonable workload was particularly evident when panel fullness exceeded 105% of the calculated panel size. Likewise, there was an inflection point between 90% and 100% of panel fullness for the association between panel fullness and burnout or intention to leave across each of the 3 years (Figure). The finding that burnout increased when panel fullness exceeded 100% of the level recommended by the VA suggests that their formula for determining panel size is well matched to the complexity of the veteran population and the PACT staffing model. Hence, efforts to improve primary care access by increasing patient panel size may prove counterproductive via increased burnout and loss of PCPs.

Empanelment differs substantially across health care systems.^21^ At an average of 1200 patients per physician PACT panel, the panel size of the VA is smaller than that for other health care systems.^21^ However, compared with the nonveteran population, the veteran population is much older and carries greater age-adjusted burdens of chronic disease, disability, smoking, obesity, chronic pain, mental health disorders, posttraumatic stress disorder, and substance use disorder.^22,23,24^ The high rates of burnout and intention to leave for VA PCPs observed in the present study may be associated with the high job demands of managing such a complex population, despite the relatively small size of VA patient panels. Meeting the needs of the veteran population may require more time during a primary care visit and more frequent follow-up visits than for the nonveteran population.

Our study identified several potential factors that may help mitigate burnout among VA PCPs. COTH membership was associated with lower rates of burnout, higher job satisfaction, and reasonable workload. This finding is consistent with observations in a survey of US physicians showing lower emotional exhaustion and greater career satisfaction among physicians with an academic affiliation and particularly those at the rank of professor.^25^ A study of inpatient VA internal medicine physicians showed that involvement in research was associated with reduced intention to leave and higher job satisfaction.^26^

Increased patient revisit rate was a second factor identified in our study that may help mitigate PCP burnout. There is strong evidence that allocation of inadequate time for primary care visits, particularly for patients with complex psychosocial needs, is associated with burnout and intention to leave.^27^ Revisits provide an opportunity to complete the care of patients with lengthy problem lists that cannot be addressed fully during a single initial or follow-up visit. Given the complexity and psychosocial needs of the veteran population, the ability to engage patients in a series of visits is likely to strengthen the PCP-patient relationship and build confidence in the PCP’s quality of care, a factor associated with protection against intention to leave and promotion of job satisfaction.^26^ We also found that a higher proportion of in-person encounters vs secure messages, video encounters, or telephone encounters was associated with decreased burnout and an increased sense of reasonable workload. This finding may indicate a benefit of in-person visits over nontraditional visits on the PCP-patient relationship.^26^

In our study, increased support staffing was associated with increased PCP burnout. This may seem counterintuitive without considering the relationship between support staffing and panel size in the VA. Increases in support staffing can trigger increases in panel size, resulting in greater workload for PCPs. Although decreases in support staffing may similarly trigger decreases in panel size, this would also increase administrative burdens for PCPs.^28,29^ Studies of the association of support staffing ratios with burnout using different methodologies have produced mixed results, with higher staffing associated with unchanged or decreased levels of burnout.^8,30^

Although our cross-sectional analysis showed a clear association between patient panel fullness and burnout, we did not observe an increase in burnout associated with higher targets for panel fullness in 2024 compared with 2023; in fact, the opposite occurred. One explanation may be that targets for panel fullness were reduced before the end of 2024, and actual increases in panel fullness were modest. Additionally, a reduction in other root causes of burnout over the same time, such as further emergence from the COVID-19 pandemic, may have mitigated the impact of increased panel fullness. Finally, sustained rates of PCP burnout above 50% may represent a ceiling above which increased job demands and decreased job resources lead not to additional burnout but instead to intention to leave.

### Limitations

There are limitations to our study. A cross-sectional design can only reveal associations, and our conclusions may not generalize beyond the VHA. Individual responses from the AES cannot be linked to individual-level panel fullness, limiting our analysis to the facility level. Hence, variability among the aggregate of facility-based PACTs may have obscured associations between individual PACT panel fullness and support staffing with outcome measures. We may have observed more associations were we able to link outcome data within each PACT to individual PACT panel fullness or staffing levels, but individual PACT team or PCP data were not available via the AES. These limitations were counterbalanced by the high response rate to the AES and the large number of PCPs included in the study. Our measure of COTH reflects historical academic affiliation and does not fully represent the extent of the educational programs, teaching roles, or trainees at facilities. Because multiple outcomes and analytic specifications were examined, some findings may reflect chance variation.

## Conclusions

This cross-sectional study found persistently high levels of burnout and intention to leave the VA in this cohort of VA PCPs. Reversing these patterns will require efforts to mitigate the root causes of burnout, such as high patient panel fullness, while enhancing factors that may help mitigate burnout.

## Notes

Supplement 1. **eTable.** Mean (standard deviation) scores for primary care provider outcomes and panel fullness by year and change in year

Supplement 2.
Data Sharing Statement
