# Supplementary material for: Panel Fullness, Burnout, and Intention to Leave Among Veterans Health Administration Primary Care Practitioners
Source: JAMA Netw Open. 2026 Sep 15;9(9):e2633671. doi: 10.1001/jamanetworkopen.2026.33671 (PMC13579432; doi:10.1001/jamanetworkopen.2026.33671)
Supplement: Supplementary file 1 [file jamanetwopen-e2633671-s001.pdf]

Supplementary Online Content

Mohr DC, Oh RC, Charness ME. Panel fullness, burnout, and intention to leave among Veterans Health Administration primary care practitioners. *JAMA Netw Open*. 2026;9(9):e2633671. doi:10.1001/jamanetworkopen.2026.33671

eTable. Mean (standard deviation) scores for primary care practitioner outcomes and panel fullness by year and change in year

This supplementary material has been provided by the authors to give readers additional information about their work.

© 2026 Mohr DC et al. *JAMA Network Open*.

eTable. Mean (standard deviation) scores for primary care practitioner outcomes and panel fullness by year and change in year [a]

Measure	2022	2023	2024	Change 2023 – 2022	Change 2024 – 2023
Burnout	.56 (.11)	.55 (.13)	.55 (.13)	-.01 (.14)	-.01 (.15)
Intention to leave	.33 (.13)	.33 (.13)	.29 (.13)	.00 (.15)	-.04 (.13)
Reasonable workload	.43 (.13)	.43 (.15)	.44 (.14)	.00 (.14)	.01 (.15)
Job satisfaction	.60 (.13)	.61 (.14)	.65 (.14)	.01 (.15)	.04 (.12)
Panel fullness	.86 (.09)	.87 (.09)	.93 (.07)	.00 (.06)	.06 (.07)

[a] Primary care practitioners include physicians, nurse practitioners, and physician assistants

© 2026 Mohr DC et al. *JAMA Network Open*.
