# Supplementary material for: Panel Fullness, Burnout, and Intention to Leave Among Veterans Health Administration Primary Care Practitioners
Source: JAMA Netw Open. 2026 Sep 15;9(9):e2633671. doi: 10.1001/jamanetworkopen.2026.33671 (PMC13579432; doi:10.1001/jamanetworkopen.2026.33671)
Supplement: Supplementary file 2 [file jamanetwopen-e2633671-s002.pdf]

Data Sharing Statement

Mohr. Panel Fullness, Burnout, and Intention to Leave Among Veterans Health Administration Primary Care Practitioners. *JAMA Netw Open*. Published September 15, 2026. doi:10.1001/jamanetworkopen.2026.33671

Data
Data available: No

Additional Information
Explanation for why data not available: Data available upon reasonable request.
